# The Angry Echo Chamber: A Study of Extremist and Emotional Language Changes in Incel Communities Over Time

**DOI:** 10.1177/08862605241239451

**Published:** 2024-03-21

**Authors:** Melissa S. de Roos, Laura Veldhuizen-Ochodničanová, Alexis Hanna

**Affiliations:** 1 Erasmus University Rotterdam, The Netherlands; 2 University of Reno, NV, USA

**Keywords:** hate crimes, internet and abuse, anything related to sexual assault, sexual assault

## Abstract

Involuntary celibates, or incels, are part of a growing online subculture. Incels are men who are unable to engage in a sexual relationship with a woman and who experience significant distress and anger as a result. In recent years, high-profile incidents of violence perpetrated by incels or those who share incel ideology have increased research attention. Incels communicate online and share several characteristics with other online extremist groups. While only a fraction of incels engage in such violence, a broader spectrum of violence should be considered, including online harassment or general violence against women. This study sought to examine how ongoing engagement on an online incel forum affects changes in incel comments in terms of expressed anger and sadness and use of incel violent extremist language. We collected comments made on an incel forum over a 3-month period. We then identified prolific users and included their comments in our analysis. To assess how their language changed, we used a text-processing program (LIWC: Linguistic Inquiry and Word Count) to assess the extent to which anger, sadness, and incel violent extremist language were expressed in the comments. Our findings indicated that incels express more anger in their comments than users on other platforms such as Reddit, Facebook, and Twitter. However, they did not express greater sadness. Further, we found that incels are already quite angry and sad when they join the forum, and they already use a fair amount of incel vocabulary. Initially, these aspects of their language increase, but they flatten over time. This pattern suggests that introduction to and embracing of incel ideology occurs elsewhere on the Internet, and prior to people joining an incel forum. Implications in terms of prevention of online radicalization and future directions are discussed.

“Incel” is a portmanteau of “involuntary celibate,” used to refer to a growing online subculture. This subculture consists mostly of young European and North American men who are incapable of engaging in a sexual relationship with a woman, despite having a desire to do so (Tastenhoye et al., 2023). Generally, they take a deterministic view to explain their negative experiences in this area: they believe that most women are only attracted to a small minority of all men, namely the most attractive and successful ones. Incels are reduced to a sexless life as a result (Scaptura & Boyle, 2020). Further, incels tend to believe that nothing they could do would improve their situation (i.e., going to the gym or engaging in self-grooming), and indeed, any attempts to do so are typically condemned by other incels (Speckhard et al., 2021). Their perceived shortcomings are viewed as being inherent and fixed, and these shortcomings result in continuous romantic rejection. These views translate into a rigid, hierarchical view of society, where incels place themselves at the bottom, desirable men (“alpha males” or “Chads”) at the top, and everyone else (“Normies”) in the middle (Scaptura & Boyle, 2020). Of greater concern, with regard to women, these views translate into misogynistic and hostile attitudes and, at the extreme end, into violent attacks that specifically target women (Green et al., 2023).

## Incel-Related Violence

In recent years, incels have received attention as a result of a few high-profile incidents of violence. These incidents have prompted security services in the United Kingdom, Canada, and the United States to recognize incels as a violent extremist threat (Moskalenko, González et al., 2022). To date, it has been estimated that roughly 50 deaths have resulted from violence perpetrated by self-identified incels (Hoffman et al., 2020; Williams et al., 2021). Although these high-profile cases made headlines, they represent only a small fraction of violent incidents that may be motivated by incel ideology.

Beyond the spotlight of media attention, many incidents remain unrecognized as being driven by this philosophy, often due to a lack of understanding or reporting (Hoffman et al., 2020). In fact, a significant proportion of violent incidents may be attributed to motivations that align with incel “ideology.” For example, although less visible and more challenging to identify, violence that is motivated by sexual frustration or loneliness should be included when considering violent incidents resulting from incels or incel-aligned ideology (Hoffman et al., 2020). Recently, Lankford and Silva (2024) have explicitly highlighted this issue that reveals that sexually frustrated mass shooters share distinct characteristics compared to other mass shooters. Specifically, they were more likely to be young, male, unmarried, and unemployed, as well as having a higher propensity for misogyny and having a higher number of female victims. With the exception of the latter, these are all characteristics that are also common among the incel community (Jaki et al., 2019). Similarly, Tietjen and Tirkkonen (2023) explored the connection between loneliness, misogyny, and radicalization, unveiling an affective mechanism known as *ressentiment* that transforms initial feelings of loneliness into hostile emotions. This broader spectrum of incel-related violence, encompassing a range of emotional underpinnings, requires closer examination.

Furthermore, it is crucial to move beyond headline-grabbing events and recognize that violence on a smaller scale may also result from incel ideology. For instance, online misogyny, a central component of incel discourse, can predict offline intimate partner violence (Blake et al., 2021). Moreover, incel-related violence may manifest in ways beyond physical violence. For example, harassment or stalking behavior directed toward individuals perceived as sexually successful should also be addressed when assessing incel violence (Schoenebeck et al., 2023). By acknowledging these multifaceted aspects of incel-related violence, we gain a more thorough understanding of the issue that will allow us to address violence within this community at its core. The primary investigative step in understanding such violence perpetration against women should be a thorough examination of these men’s emotional state, particularly in terms of anger and sadness, given its established association with an elevated risk of both intimate partner violence and nonpartner sexual violence (Ramsoomar et al., 2023; Yu et al., 2019).

### Incels’ Emotional Experience

Increasingly, we learn about the emotional experience of incels, a critical aspect of comprehending the complex dynamics within this online subculture. Recent research, such as that by Costello et al. (2022), has begun to shed light on the psychological challenges faced by some incels. This study, involving 151 self-identified incels, revealed a striking contrast when compared with similar-age nonincels. Incels reported a greater tendency for interpersonal victimhood—a psychological phenomenon wherein individuals perceive themselves as victims and often incorporate this perception into their identity. This victimhood mindset is a precursor to the radicalization process, often stemming from perceived marginalization (Lyons-Padilla et al., 2015). Additionally, incels reported significantly higher levels of depression, anxiety, and loneliness than nonincels, each of which may contribute to feelings of both anger and sadness.

This anger and sadness is evident on incel forums. Users often openly share their emotions and, in many cases, even share thoughts of suicide with each other online (Baele et al., 2019; Daly & Laskovtsov, 2021; Hoffman et al., 2020; Jaki et al., 2019; Williams & Arntfield, 2020). Alarmingly, an incels.co survey in 2020 found a staggering 82.3% of incels reported having considered suicide (Incels.co, 2020), underscoring the severity of the struggles experienced by this group of individuals. All of these factors collectively converge within the category of sadness, making it a pivotal focal point when examining the psychological experiences of this group.

These psychological challenges can be attributed to a variety of factors such as romantic rejection, experiences of bullying, or loneliness. Romantic rejection, although a profound source of distress for many individuals, can take a particularly heavy toll on those who internalize these experiences and attribute their difficulties to inherent personal flaws (Sparks et al., 2023). Such self-blame may foster feelings of inadequacy and hopelessness, pushing them toward online forums where they may find others who share similar experiences, such as those within the incel community (Broyd et al., 2022). Furthermore, as found by Moskalenko, González et al. (2022), incels often report experiences of bullying and persecution. Individuals may then find validation and camaraderie among individuals who have faced similar forms of social ostracization. The incel ideology, with its narratives of societal rejection and mistreatment, may resonate deeply with those who have experienced bullying, possibly leading them to take interest in this online community and, in turn, to adopt their beliefs (Moskalenko, Kates et al., 2022). Moreover, loneliness can serve as a gateway to becoming acquainted with this subculture as users may seek comfort in shared experiences (Sparks et al., 2023). However, as they further engage in discussions and become immersed within the ideology, their sense of belonging may strengthen, further enhancing their isolation from the “outside world” (Daly & Reed, 2021). Although such factors can contribute to an individual’s involvement within this community in isolation, they often intersect and reinforce one another. For many, the incel community serves as a refuge from these emotional burdens, offering a sense of belonging and shared identity that can be enticing in the face of their struggles.

Although sadness is a prominent emotion among many incels, another prevalent sentiment that characterizes this community is anger. Anger often emerges along a similar pathway as sadness from incels’ shared grievances, experiences of bullying, and feelings of societal rejection (Lindner, 2022). Although the community is diverse in the sense that not all members endorse violence, a subset does express their anger in a more extreme form. This has been demonstrated by studies such as that of Speckhard et al. (2021), who found that an increase in self-reported misogyny was linked to a greater willingness to engage in violence. Strikingly, this increase in misogyny was also linked to an increased agreement with the statement: “I would rape if I could get away with it.” Such findings underscore the urgent concern in addressing not only the prevalence of misogyny within the incel community, but also the underlying anger associated with it, and a broader range of violence perpetrated by incels. On the one hand then, online incel communities may provide support and a sense of belonging that may alleviate sadness and anger. On the other hand, exposure to and engagement with such online communities may also lead to an escalation of negative affect and increasingly hostile views toward the perceived out-group.

### Online Radicalization of Incels

Online radicalization within the incel community is a complex phenomenon, which is distinct from overtly political radicalization. It warrants scrutiny due to the extreme views held by certain incels, which seem to signify a desire to overthrow society and subjugate women, often through a lens of misogyny (Hoffman et al., 2020). Specifically and alarmingly, studies have exposed the disconcerting level of support for violence against women within this community (Baele et al., 2019), with some incels openly advocating for such violence (Jaki et al., 2019). This endorsement of violence can be regarded as a form of radicalization, where the aim is to disrupt established norms and societal structures. This deep-seated misogyny, further reinforced by online echo chambers and peer support, often drives them toward adopting increasingly extreme ideologies, with violence becoming a means of retribution and control (O’Donnell & Shor, 2022).

Several researchers have drawn parallels between incels and other extremist groups. Like other extremist factions, incels form connections with like-minded individuals primarily through online platforms, eliminating the need for physical proximity. This online connectivity fosters an environment where they can reinforce their beliefs and grievances, creating a self-reinforcing echo chamber (Baele et al., 2019). Moreover, through the process of indoctrination, new members are introduced to the group’s ideology and gradually become desensitized to radical ideas, hostile language, and violence toward out-group members (Soral et al., 2018; Williams & Tzani, 2022). Additionally, a subset of incels appears to glorify recent acts of violence, further blurring the lines between incel ideology and extremist behaviors (O’Donnell & Shor, 2022). Nevertheless, unlike many other extremist groups, incels lack a shared objective or utopian vision, which may have implications for their propensity for violence (Pelzer et al, 2021). The absence of a unifying goal could result in less organized and coordinated violence, making their attacks more challenging to anticipate.

Moreover, the concern surrounding the online radicalization of young men introduces an additional layer of complexity to this issue. Vulnerable individuals, particularly those grappling with isolation and detachment from society, may unwittingly become ensnared by extremist ideologies. This issue is evermore exacerbated by Internet algorithms that progressively lead them toward increasingly harmful content (Helm et al., 2022; Mamié et al., 2021). This issue has been illustrated by the story of Reid Brown, a young boy who was exposed to extremist material at the age of 13 (Wilson, 2022). In this news article, Reid Brown vividly recalls his experience watching YouTube videos, which gradually began offering more and more extremist and controversial content. The consequences of such online influence became evident in his day to day life when he and his friends began echoing the sexist attitudes they had encountered online. Considering such cases, it is essential to recognize the allure of these online communities for those seeking belonging and identity. These young men may inadvertently discover these groups, offering them a newfound sense of community and, alarmingly, a misguided source of empowerment (Joint OSCE Secretariat—OSCE ODIHR, 2013).

One significant vulnerability within this subculture is the concept of interpersonal victimhood, which entails individuals perceiving themselves as victims and subsequently making victimhood a core component of their identity. In the case of incels, this interpersonal victimhood is often tied to their masculinity and inability to form sexual and romantic relationships (O’Malley & Helm, 2022; Speckhard et al., 2021). This perception of marginalization and victimization experienced by incels can serve as a precursor to the radicalization process, aligning closely with the initial stages of various theories of online radicalization (Neo et al., 2017).

Although only a small portion of this community actually commits acts of violence, the potential for radicalization and the endorsement of violence within the larger incel community remain significant concerns. For instance, the social support from other incels in an online forum may push one extremist incel from fantasizing about violence and describing these fantasies in the forum to actually committing an act of violence in real life. Addressing these issues requires a multifaceted approach that not only includes identifying and intervening with those on the brink of radicalization, but also addressing the root causes of misogyny and victimhood within this community.

### Present Study

To date, what we know about Incels primarily comes from Internet data, surveys, or interviews conducted with self-identified incels at a single time point. However, while sadness and anger have been assessed through these methods, and violent extremist incel language has been identified at a platform level in online incel forums, we do not know how individual users’ language and emotion expression changes during their engagement on an incel forum. As such, this study seeks to examine how prolonged interactions on an incel forum affect users’ language in terms of affective tone (anger and sadness) and use of extremist language. We expect users’ language to become more angry, sad, and violent-extremist with longer interaction on the forum.

## Methods

### Data Collection and Preparation

Data were collected from the public section of the most prolific Incel forum (https://www.incels.is). Using Beautifulsoup and Requests in Python, we webscraped 100 pages, each containing 100 forum posts on the last day of March 2022. For each comment, we saved the username, user join date, number of posts made by user, user’s popularity score, title of the thread the comment was made in, timestamp of the comment, and the comment itself. The number of posts and popularity score were dynamic and reflected values at the time of the webscrape. We then isolated all comments made between January and March of 2022, which comprised a total of 135,729 comments. Following this, we included all comments made by users who had commented at least 100 times in at least 2 of the 3 months. This led to an inclusion of 166 unique users.

We then conducted a rudimentary spellcheck to remove typos and write out common abbreviations (i.e., jsyk = just so you know; tbh = to be honest, etc.). Blank comments were removed, as well as any quoted text users replied to. Finally, as per LIWC recommendations, we removed all comments of less than 10 words. This process still left a large range of data points, so we collated comments made per week, so that each user had a maximum of 12 data points (one per week for the full 3-month period).

### Ethical Considerations

In their 2022 article, Luscombe et al. (2022) discuss various ethical considerations to take into consideration when conducting a web scrape. The first is harm to the website. The web scrape was conducted in such a manner that the website was not overburdened and at risk of crashing through limiting the frequency of demands and conducting the scrape more slowly. Further, the authors contend that ethics in the area of automated text processing from publicly available online data remains a moving topic. In the case of incels, we argue that this is a particularly difficult to reach population, which forms a concern both in terms of online radicalization of young people and the potential of violence motivated by incel ideology. In this sense, a “greater good” argument can be made for the web scraping. Further, we used participant numbers rather than user IDs to link data, and only aggregate data were used in this study, which makes it impossible to trace any findings back to an individual user.

### Procedure

The data were compiled in one excel spreadsheet with all variables scraped, the collated comments per week, and the added variable of week sequence. We used LIWC (Boyd et al., 2022) to analyze the nature of the language used in our dataset. LIWC is an automated data-processing software that allows large bodies of text to be compared against prevalidated dictionaries on a broad range of topics. Centered around the idea that our psychological states are reflected in the language we use, the current LIWC dictionary comprises more than 12,000 words over many subdictionaries. The LIWC dictionaries were created from a corpus totaling more than 31 million words, across a variety of sources. LIWC has been used extensively to analyze large bodies of text in various forms (for a review, see Boyd & Schwarz, 2021).

Each unit of analysis (in this case, each collated comment cell) is compared to the selected dictionaries, and a percentage of how many words match dictionary entries is provided. This percentage serves as the score, or metric of analysis, for each variable (i.e., sadness, anger, extremist language). For example, a comment with 0.5% overlap in “sadness” words is assigned a score of 0.05, which can then be compared to another comment assigned a 0.1 on sadness, representing 0.1% overlap with sadness words in the dictionary.

Further, LIWC provides norm groups across various sources to compare output to. For example, LIWC provides data from Facebook and Twitter as norm metrics for typical percentages of word use according to different dictionaries. This norm system allows us to compare sadness, anger, and other word percentages in the incel comments to typical percentages found on other less extremist platforms.

LIWC recommends using the Kuder-Richardson Formula 20 (KR-20), which should be thought of as a more accurate measure of internal consistency than Cronbach’s alpha for language categories (Boyd et al., 2022). This is due to the highly variable base rate of word usage, which Cronbach’s alpha is less equipped to handle, since it uses relative word frequencies. KR-20 on the other hand, estimates reliability in a binary (absent or present) manner. The LIWC psychometric manual (Boyd et al., 2022) provides KR-20 values for the dictionaries developed by LIWC.

### Materials

#### LIWC—Summary Variables

LIWC calculates several summary variables from the data it processes. For the purpose of this study, we included: word count, words per sentence, analytical thinking, clout, and authenticity. Analytical thinking assesses the extent to which people’s choice of words reflects formal, logical thinking patterns (Pennebaker et al., 2014). People who score low on this dimension are more likely to use intuitive language and come across as friendly, whereas high scores have been linked with academic achievement and reasoning skills, but also perceived coldness and rigidity. Clout is an indication of social status as displayed through language (Kacewicz et al., 2014). It builds on the work of Becker (1963), who held that (deviant) subcultures share distinct values and cultural practices, which affect individuals’ self-concept. Becker (1967) coined the term “hierarcharchy of credibility,” to explain it is those at the top of the hierarchy have the credible view of how things work. In the context of incels, who in a broader sense identify as at the bottom of the societal hierarchy, while strictly enforcing hierarchy within their own ranks, this construct seems particularly salient. People whose language has a higher level of clout are more concerned with others, whereas those who display lower clout tend to be more focused on themselves. Finally, authenticity reflects the extent to which people use language freely, with low scores indicative of high self-monitoring (Newman et al., 2003). Authenticity is about being one’s true self and can be characterized as a self-reflective emotional experience (Vannini & Franzese, 2008). In an online community that has strict requirements regarding membership of that community, authenticity is likely to come under pressure from other members, and the need to adjust to the values of the community in order to fit in may lower authenticity in incel speech.

#### LIWC—Anger Dictionary

To assess anger in the data, we used the LIWC-provided Anger dictionary (Boyd et al., 2022). It consists of 181 words, such as “hate,” “mad,” “angry,” and “frustrated.” It has good internal reliability, with a reported KR-20 of .82.

#### LIWC—Sadness

We used the LIWC-provided Sadness dictionary to assess the presence of sadness in the data. This dictionary consists of 134 words such as “sad,” “disappoint*,” and “cry.” It has good internal reliability, with a reported KR-20 of 0.80 (Boyd et al., 2022).

#### Incel Violent Extremism Dictionary

We assessed the use of violent extremist language of incels using this recently developed dictionary. This dictionary was developed based on an extensive corpus of several online spaces that are part of the incelosphere (Baele et al., 2023). It comprises 174 words, along three broad dimensions: (a) verbs that express acts of violence (e.g., “stab,” “kill” or “rape”), (b) nouns that label weapons (e.g., “gun,” “knife,” or “acid”), and (c) nouns that dehumanize members of the out-groups (e.g., “femoids,” “roasties,” or “curries”).

## Results

### Sample Characteristics

Descriptive statistics of users and comments are displayed in Table 1. There were wide discrepancies in how long people had been active on the forum, and how active they were during this time. We compared the three summary variables with the norm groups provided by LIWC for Facebook (FB), Reddit (R), and Twitter (TW). Our sample’s comments had lower scores on analytic, higher scores on clout and lower authenticity scores (see Table 2). This suggests that compared to users on other social media platforms, incels express themselves less logically, with a greater focus on others than the self, and with greater self-monitoring. The higher score on clout underlines that despite their low moral status in society, incels concern themselves with enforcing hierarchies within their own ranks, as reflected in their language. Further, lower authenticity hints at a need to fit in and modify one’s language to meet the standards and requirements of the subculture.

**Table 1.** Descriptive Statistics of User Characteristics.

Variable	*M* (*SD*)	Min–Max
Time since join date	1 year, 7 months, 17 days (1 year, 4 months, 11 days)	1 month, 8 days – 4 years, 4 months, 22 days
Number of posts	6,774.48 (13,322.20)	131–116,000
Popularity	3.74 (1.43)	1–5
Word count/week	602.76 (793.36)	10–6,037
Words/sentence	44.12 (61.78)	1–739

**Table 2.** Descriptive Statistics of Summary and Dictionary Variables in Incel Sample and Compared with Norm Groups.

	Incels.is		Norm Groups *M* (*SD*)		
Variable	*M* (*SD*)	Min–Max	Twitter	Facebook	Reddit
Analytic	44.12 (61.78)	1–99	42.86 (27.48)	47.06 (18.22)	35.21 (16.03)
Clout	41.84 (26.89)	1–99	49.10 (28.36)	38.74 (22.03)	31.40 (19.14)
Authenticity	43.61 (26.46)	1–99	52.33 (25.58)	62.38 (22.58)	61.74 (20.09)
Incel violent extremism dictionary	1.53 (1.41)	0–10	Na	Na	Na
LIWC anger	.28 (.55)	0–8.33	0.18 (0.20)	0.22 (0.23)	0.19 (0.25)
LIWC sadness	.16 (.39)	0–18.75	0.17 (0.23)	0.39 (0.51)	0.14 (0.23)

Next, we examined the descriptive statistics of our three chosen dictionaries, which are displayed in Table 2. Note that norm groups have not (yet) been provided for the incel violent extremist dictionary (IVED). In terms of anger, we see the Incel comments score higher than norm groups on all other social media platforms. A different picture emerges for sadness, with incel comments scoring similar to posts on Twitter and Reddit, but Facebook users showing the highest levels of sadness in their language. In other words, incel users tend to use more angry language than users on other social media platforms, but they do not tend to use more terms reflecting sadness.

### Language Use and Join Date

To test whether users had different anger, sadness, and IVED language use based on when they joined the platform, we first calculated each person’s median score on these three variables across weeks. We chose to examine the median score because the percentage scores calculated from the LIWC analyses were fairly skewed due to the high amount of “0” scores assigned for mundane comments, so the median was generally a better reflection of the person’s “typical” language use in nonmundane comments than the mean.

The correlations between each person’s median anger, sadness, and IVED percentage scores with join date are presented in Table 3. Users’ join date on the online incel forum was positively related with their median levels of anger, sadness, and incel violent extremist language, indicating that users who joined more recently had the highest percentages of these terms in their comments on the forum. Intriguingly, anger language use was positively related to incel terminology use, whereas sadness was not.

**Table 3.** Relationships Between Forum Join Date, Anger, Sadness, and Incel Extremist Language.

Variable	1	2	3	4
1. Join date	—			
2. Anger	0.17*	—		
3. Sadness	0.21**	0.20*	—	
4. Extremist language	0.21**	0.24**	0.05	—

*Note*. All language variables reflect each person’s median score on that variable across 12 weeks of comments on the forum.

* *p* < .05. **p < .01.

### Changes in Language Use Across 12 Weeks

Finally, we assessed whether participants’ language changed over the course of 12 weeks. To quantify these patterns and estimate the slopes of change, we specified growth models for anger, sadness, and IVED language scores for all participants over a 3-month span. First, we tried modeling both *linear* and *nonlinear* (i.e., quadratic) patterns of change across all 12 weeks for each variable. However, due to the high model complexity of estimating parameters with this many data points and the high degree of variability in change patterns across participants, several models either had poor model fit or did not converge at all (i.e., failed to reach a single set of parameter estimates). Thus, we present slightly different variations of the growth models for each variable. Specifically, the results of the best-fitting model for each of the three variables are presented in Table 4. By using the best-fitting model, the results are most interpretable according to the specification that is the closest match to the actual change patterns in the data.

**Table 4.** Growth Models for Within-Person Changes in Anger, Sadness, and Incel Extremist Language Use Over 12 Weeks.

Model	Model Fit: CFI, RMSEA, SRMR	Intercept	Linear Slope	Quadratic Slope
Anger	1.00, 0.00, 0.00	0.16**	0.06**	−0.01**
Sadness	0.14, 0.04, 0.16	0.13**	0.01	—
Incel Lang. (IVED)	0.97, 0.03, 0.09	1.13**	0.14**	−0.01**

*Note. N* = 166 incel forum users. All models estimated growth (i.e., changes) in language scores across 12 weeks on the forum, but in different ways. The Anger model estimated a quadratic growth model with changes across Weeks 2, 5, 9, and 12. The Sadness model was a linear growth model across all 12 weeks of data, but no growth models for Sadness fit the data well. The IVED model estimated a quadratic growth model across all 12 weeks of data. IVED = incel violent extremist dictionary.

* *p* < .05, ** *p* < .01.

First, the growth models for anger scores using all 12 weeks of data did not fit the data well. This misfit could indicate that (a) change was not happening, (b) there were many increases and decreases across weeks, so a growth pattern was not consistent, or (c) there was substantial variation in change patterns across participants, so the model could not accurately specify average change patterns. To estimate the change pattern at the week level, we next specified a latent basis model, which estimated the slope loadings and proportion of change in anger scores happening in each week. This model showed that there was a dip in scores from Week 1 to Week 2, but otherwise scores seemed to increase across weeks.

Thus, to capture this change pattern and reduce model complexity, we specified linear and nonlinear growth models starting in Week 2 (i.e., dropping Week 1), and we only used four data points to assess changes at the month level: Week 2, Week 5, Week 9, and Week 12. The nonlinear (i.e., quadratic) growth model for these anger scores allowed scores to follow a nonlinear trajectory, and this model had excellent fit to the data (χ^2^ = .45 [*p* = .50], comparative fit index (CFI) = 1.00, Tucker-Lewis index (TLI) = 2.24, root mean square error of approximation (RMSEA) = 0.00, standardised root mean square residual (SRMR) = 0.00). The average intercept was significant (*i* = 0.16, *p* < .01), indicating that users had significant anger scores starting in Week 2. Additionally, both the linear slope (*s* = 0.06, *p* < .01) and quadratic slope (*s*^2^ = −0.01, *p* < .01) were significant, indicating that on average, anger trajectories increased over time (i.e., positive linear slope estimate), but then leveled off somewhat (i.e., negative quadratic slope estimate).

For sadness, we tried a linear growth model, a quadratic growth model, and a latent basis model in which the time pattern was freely estimated, but none of the growth models fit the data well. Similar to the process for the anger models, we investigated the change patterns at the week level more closely with the latent basis model, and there were many inconsistent patterns of increasing and decreasing scores, indicating that it would likely be difficult to estimate a change pattern overall in any meaningful way. We also tried estimating models with less data points in a similar way as anger, but the models still did not fit well.

We presented the results of the linear growth model using all 12 weeks of data, but given the generally poor fit (χ^2^ = 6.02 [*p* = .31], CFI = 0.14, TLI = -0.04, RMSEA = 0.04, SRMR = 0.16), they should be interpreted with caution. Again, the average intercept was significant (*i* = 0.13, *p* < .01), indicating that users had significant sadness scores starting in Week 1. However, the linear slope (*s* = 0.01, *p* = .74) was not significant, indicating that on average, sadness trajectories were not significantly different from 0.

Finally, we specified linear and nonlinear growth models for IVED scores, and the nonlinear model using all 12 weeks of data fit the data well (χ^2^ = 79.23 [*p* = .19], CFI = 0.97, TLI = 0.97, RMSEA = 0.03, SRMR = 0.09). The results of this model are presented in Table 4. The average intercept was significant (*i* = 1.13, *p* < .01), indicating that users had significant IVED language scores starting in Week 1. Additionally, both the linear slope (*s* = .14, *p* < .01) and quadratic slope (*s*^2^ = -0.01, *p* < .01) were significant, indicating that on average, IVED trajectories increased over time (i.e., positive linear slope estimate), but then leveled off somewhat (i.e., negative quadratic slope estimate), similar to the anger patterns. Notably, across all three variables, the parameter estimates are largest for IVED. In other words, the percentage scores estimated from LIWC were highest initially (average intercept of 1.13% IVED language in Week 1) and increased at the highest rate (average increase of 0.14% per week).

## Discussion

The aim of this study was to examine how the language of individual incels develops as they spend more time on an incel forum. Through the analysis of comments, we assessed changes in anger, sadness, and use of incel violent extremist language. We found that, compared with norm groups from Twitter, Facebook, and Reddit, incels displayed high levels of anger in their online communications. This observation aligns with previous research indicating that many incels commonly harbor a deep-seated anger and resentment toward women as a whole, coupled with a sense of marginalization stemming from societal norms and expectations (Jaki et al., 2019; Speckhard et al., 2021). These prevalent factors within the incel community likely contribute to the heightened levels of anger evident in forum discussions.

Additionally, we found that sadness expressed in incel comments was similar to norm groups from Twitter and Reddit, but much lower than Facebook. This discrepancy may be attributed to the differing patterns of emotional expression within these platforms. Facebook, as a predominantly social and personal sharing platform, may encourage users to express a wider range of emotions, whereas Twitter and Reddit often involve more public and topical discussions, leading to less frequent expressions of emotions (Guimarães et al., 2019). Incel forums, on the other hand, typically focus on discussions related to specific grievances, shared experiences, as well as discussions related to frustration and anger (Jelodar & Frank, 2021). Such topics could, in turn, overshadow expressions of sadness leading to the observed differences between the two platforms.

Furthermore, we found that incels initially showed an increase in their use of anger and IVED language, which leveled off in later weeks, consistent with a quadratic growth model. This finding is consistent with aspects of social norms and group dynamics wherein newcomers feel the need to adapt to the norms and expectations of the community (Harwood, 2020). The initial surge in anger and extremist language usage may be an attempt to establish their identity and conform to the group’s expectations, potentially driven by a desire for social acceptance. Over time, as users become more accustomed to the forum’s norms, the need to maintain this heightened level of expression may diminish. This leveling-off effect may suggest that incels’ language usage could be influenced by both external pressures to conform as well as the evolving normative climate within the community (Crandall et al., 2002).

Moreover, our study revealed a significant positive correlation between the Anger and IVED scores. This link between heightened anger and the increased use of extremist language indicates that anger may act as a potential precursor or catalyst for adopting such rhetoric, and vice versa. The implications of this finding become more pronounced when considering its resonance with broader issues, particularly in the context of violent incidents. Notably, many mass shooters, some of whom have been associated with incel communities, exhibit marked levels of intense anger, underlining the need to closely monitor and address anger as a potential warning sign to more extreme forms of hostility within this community (Lankford & Silva, 2024). This pattern is echoed by a study examining an alt-right online group, showing a linear increase in anger and a subtle rise in hate speech; both behaviors were marked as warning signals (Grover & Mark, 2019). However, it is important to exercise caution in interpreting this finding, as the correlational nature of the link does not definitively establish the direction of the relationship.

Conversely, it was found that the IVED showed no significant association with sadness. This intriguing finding suggests a dissociation between the expression of anger and the expression of sadness within the incel community. One possible explanation for this distinction could be rooted in the motivational differences between these emotions. Anger often involves a sense of injustice, perceived threats to one’s status or identity, and a desire for action or retribution (Kim, 2009; Silva, 2021). In contrast, sadness may be more closely tied to feelings of hopelessness, powerlessness, or resignation (Newman et al., 2023; Sanderson et al., 2020; TenHouten, 2016). In the context of incel forums, where resentment and a desire for change are prevalent, the emotional energy may be channeled into anger-fueled expressions, whereas sadness, being perceived as a more passive emotion, may not lend itself to the violent and extremist language associated with the IVED.

Interestingly, this study revealed that incels displayed heightened levels of each of the three variables shortly after joining the forum. This suggests that the individuals who become part of these online communities may already bring with them a considerable degree of anger and sadness, as well as some knowledge about the incel community and the specific language they use. This finding hints at the possibility that incels are not necessarily radicalized within these forums, but rather join them with preexisting emotional turmoil. This notion is supported by research conducted by Champion and Frank (2021) which explored the radicalization pipeline on YouTube. This study revealed that radicalization activities on widely accessible platforms such as YouTube facilitate both the passive and active consumption of extremist ideologies. Given that “misogynist terrorism” has been observed to fuel instances of mass violence, the existence of a radicalization pipeline for incel-related content underscores the urgency of monitoring and addressing this issue at earlier stages, even in nonincel specific social media sites. Understanding the emotional states of individuals before they engage with these communities could help tailor interventions aimed at preventing the radicalization of incels. One possible avenue to explore could be to review internet algorithms and attempt to curb the virality of extremist incel content.

An alternative explanation for the initial increase in the three variables could be attributed to the evolving experiences of users within the community. It is plausible that users who remain active on the forum for an extended period may find the social support and camaraderie that led them to join the forum in the first place. Even if support was not their primary objective for joining, they may still encounter it as they spend more time on the forum. This emerging group identification could mitigate their initial emotional distress leading to the leveling off of these variables in later weeks. A possible avenue for future research could involve investigating the dynamics of group identification within the incel community and whether this indeed increases over time. It is crucial to acknowledge, however, that the relationship between group identification and extremist behavior is complex and raises its own set of challenges. This complexity is illustrated by Cohen et al. (2014) who found that indicators of increased group identification such as heightened emotional responses to events affecting the in-group or increased linguistic similarities to other radical role models may, in fact, be a warning behavior for radical violence. Thus, although prolonged group identification may have a mitigating effect on the initial surge of emotions, it is important to note that it may also signify an increased risk of radicalization, warranting careful scrutiny.

In addition to the complex nature of group identification within this subculture, another pressing concern is the association between incel communities and deviant sexual interests, such as the large amount of support for pedophilia as highlighted in a recent report (Center for Countering Digital Hate, 2022). Alarmingly, this report revealed that over half of the users on the incels.is forum expressed support for pedophilia and over a quarter of users posted words associated with pedophilia such as “loli” and “jb” (short for jailbait), both of which refer to girls who are under the age of consent. Although this study does not directly address the topic of pedophilia, it does emphasize the emotional distress that individuals may bring when joining these communities and how this may lead to a stronger group identification and thus most probably also a stronger acceptance of extremist ideologies. Addressing these factors and uncovering the emotional dynamics underlying these issues is, therefore, a crucial area to explore in future research.

The results of this study have several practical implications. First, algorithms on “mainstream” platforms should be scrutinized with more attention paid to how these direct young men who experience romantic loneliness and rejection toward incel rhetoric. In a similar vein, there is a need for accessible resources for those who spend a significant amount of time online, in isolation, and who are experiencing negative affect. Indeed, mental health resources or emotional resilience programs should be integrated into online platforms such as YouTube and X (formerly Twitter). By making such resources easily accessible, and equipping young men with more adaptive coping mechanisms to manage their negative affect, the chances of them seeking such support on incel forums may be reduced. Second, while incels are not likely to seek therapy or professional support for their struggles, mental health professionals should increase their understanding of how feelings of loneliness and anger operate in young men who are disappointed by the dating world, and they may further probe for online behaviors. Early detection and intervention of escalation of misogynistic beliefs seems both relevant, and such an escalation may be more widespread than generally assumed (Townsend, 2022).

### Strengths and Limitations

One notable strength of this study is the integration of both qualitative and quantitative data. By incorporating the actual comments of the users, we were able to gain a rich, nuanced understanding of language and emotional dynamics within this forum. The use of qualitative data allows for an in-depth exploration of their expressions, whereas the quantitative analysis provides a systematic approach to assess the content of the language. Nevertheless, this type of analysis does not take into account cultural artifacts, norms, and mores, which limits the depth of understanding gleaned from the text analysis.

Another compelling strength of this study is the temporal perspective employed in examining how extremist groups interact and adapt language use over time. By tracking the changes in language patterns across a 12-week period, we were able to gain a deeper understanding of how language evolves at an individual level within the incel community over an extended period of time. This provided valuable context for assessing the factors contributing to the group’s identity and the adoption of extremist ideologies. Similarly, this temporal dimension allows for a more comprehensive analysis of the emotional trajectories of users within this forum and sheds light on how these communities function and change over time.

When interpreting the findings of this study, several limitations should be considered. First, this study used an arbitrary 3-month time frame for data collection. This decision was necessitated by technical constraints and thus may not fully capture the long-term evolution of language patterns within this community. Although the study assesses change over time, the findings are indicative of relatively short-term changes as many individuals are members for a longer period of time than 3 months. Thus, in light of this limitation, future research in this area should aim to employ more extensive and precise data collection methods. This could be done by studying new users from the moment they join the forum and observe how their language and emotional expressions evolve over an extended duration. This would provide a more comprehensive understanding of the long-term adaptation processes within the community, shedding light on the potential escalation or de-escalation of extremist language use.

Second, the presence of nonincel-related topics and other noise in the data needs to be addressed. Many comments were not exclusively focused on incel-related discussions, which may have led to a dilution of the percentage estimates. As a result, the observed patterns may underestimate the true linguistic tendencies and emotional dynamics of the users on this forum. Future research efforts should therefore consider focusing solely on extreme posts, which are likely to provide a clearer indication of behavioral tendencies and the risk of violence within this community. By concentrating on the most concerning content, future studies can provide a more accurate assessment of the relationship between language usage and the risk of extremist actions.

Third, LIWC is a valid but crude measure that relies on frequencies rather than context. However, LIWC takes advantage of probabilistic models of language use (Boyd et al., 2022), which minimizes the risk of incorrectly elevating scores. Further, we collated comments to ensure that each unit of analysis consisted of a sufficient number of words, which further reduces the likelihood of such counting “errors.” Finally, while the website has strict rules about only allowing incels to post on their forums, and any suggestion that someone is faking at being an incel is investigated and may result in a ban, it is possible that “trolls” or fakers were part of this sample. However, given that we included only users who were prolific over a 3-month period, we can assume with some confidence that the users in our sample indeed self-identified as incels.

## Conclusion

The examination of the linguistic dynamics within the incel community is a critical step in understanding the emotional underpinnings of this subculture and their potential for online radicalization. This study examined how prolonged interactions on an incel forum affect users’ language in terms of affective tone (anger and sadness) and the use of extremist language, aiming to shed light on the evolving emotional dynamics that underpin this community. Our findings revealed that incels exhibited elevated levels of anger compared to other social media platforms. Additionally, a correlation was found between anger and the use of violent and extremist language, suggesting that anger may serve as a precursor to adopting extremist rhetoric. Furthermore, the findings suggest the possibility that individuals who join the forum often bring with them preexisting emotional distress in the form of anger and sadness, which is reflected in their forum posts. This study highlights the need for early monitoring and intervention, not only within incel-specific platforms but also across other more general social media sites like YouTube or Twitter. Future research should aim to understand the emotional aspects of individuals before their involvement with these communities as this is essential in countering the spread of extremist incel content and mitigating the chances of individuals becoming radicalized online.
